# The clinical characteristics and treatment response of myasthenia gravis with positive thyroid peroxidase antibody or thyroid globulin antibody

**DOI:** 10.1016/j.jtauto.2025.100328

**Published:** 2025-10-29

**Authors:** Hanyu Xin, Mingou Lu

**Affiliations:** Neurology, Dalian Medical University, Dalian, China

**Keywords:** Myasthenia gravis, Thyroid peroxidase antibody, Thyroglobulin antibody

## Abstract

**Background and purpose:**

Myasthenia gravis (MG) frequently associates with thyroid peroxidase antibody (TPOAb) and thyroglobulin antibody (TgAb), indicating shared autoimmunity. This study investigated the impact of TPOAb/TgAb on MG severity and pathogenesis, to inform improved management.

**Methods:**

We retrospectively analyzed 144 MG patients (Jan 2022–Dec 2024), stratified into four groups by TPOAb/TgAb status. We compared demographic and clinical profiles, analyzed the effects of disease duration, stage, and onset age on TPOAb/TgAb titers, and evaluated treatment responses across groups.

**Results:**

Among 144 included patients, 55.6 % were thyroid antibody (TAb) - positive (TPOAb + TgAb-: 18.8 %; TgAb + TPOAb-: 9.7 %; TPOAb + TgAb+: 27.1 %). Median onset age was 61 years, 67.5 % were female. TPOAb + TgAb- MG primarily presented as MGFA I (70.4 %), initial onset (63.0 %), with low thymoma (3.7 %) and no TitinAb/RyRAb. TPOAb + TgAb + MG exhibited a severe phenotype: higher MGFA III (30.8 %), relapsed (71.8 % vs 50.0 % in TPOAb-TgAb-, P = 0.030), thymoma (43.6 %), and TitinAb/RyRAb positivity (43.6 %/25.6 %). TgAb titers were significantly higher in relapsed MG (P = 0.001). Both TPOAb + TgAb+ and TPOAb-TgAb- MG responded better to pyridostigmine 90 mg/day, TPOAb + TgAb + MG showed superior response to high-dose glucocorticoids (40–60 mg/day) compared to TPOAb-TgAb- (P = 0.006). However, multivariate analysis indicated TPOAb + TgAb + status itself was not an independent predictor (OR = 0.077, 95 % CI: 0.000–23.487; P = 0.380).

**Conclusions:**

This study demonstrates the clinical significance of TPOAb/TgAb in MG. TPOAb + TgAb + status identifies a clinical subgroup with a "triple-high" profile. TgAb may show potential as a disease activity biomarker. These findings inform precision treatment strategies, pending validation in large prospective studies.

## Graphical abstract

**Figure:**
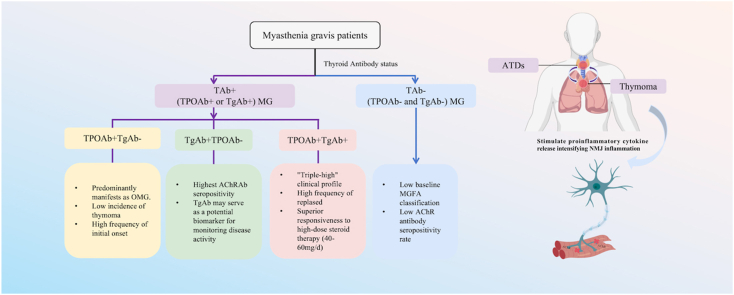

## 1 Introduction

Myasthenia gravis (MG) is an acquired autoimmune disorder mediated by pathogenic autoantibodies that impair postsynaptic membrane signaling at the neuromuscular junction (NMJ), clinically characterized by fatigability and fluctuating skeletal muscle weakness. Pathogenic antibodies targeting acetylcholine receptors (AChR) (IgG1/IgG3 subtypes) are detectable in 80–85 % of MG patients [1]. Among the remaining cases, 5–10 % are driven by anti-muscle-specific kinase (MuSK) antibodies (IgG4 subtype), while less than 2 % harbor antibodies against low-density lipoprotein receptor-related protein 4 (LRP4). Notably, although antibodies against titin and ryanodine receptors (RyR) are not directly pathogenic, they demonstrate significant associations with disease severity and prognosis in thymoma-associated MG [2]. Epidemiological evidence from Europe demonstrates a progressive increase in both the incidence and prevalence of MG in recent years, particularly among elderly populations [3]. Global data indicate an approximate mortality rate of 5.9 %, primarily attributed to myasthenic crisis and respiratory complications [4,5]. Chinese epidemiological studies report an incidence rate of 0.68 per 100,000 population with a female predominance (male-to-female ratio 1:1.5) [6], consistent with data from other Asian regions. Despite continuous improvements in diagnostic and therapeutic approaches for MG, its precise pathogenic mechanisms remain to be fully elucidated.

Clinical epidemiological data reveal that MG patients frequently develop comorbid systemic autoimmune disorders, with autoimmune thyroid diseases (ATDs) showing the most prominent association (comorbidity rate: 15–30 %). This significant disease coexistence likely stems from their shared organ-specific, antibody-mediated autoimmune pathogenesis [7,8]. Extensive evidence has established the crucial involvement of thymic abnormalities in MG pathogenesis. Rotondo et al. [9]demonstrated that the thymus not only plays a pivotal role in MG development but may also contribute to maintaining thyroid autoimmunity, with thymic microenvironment alterations potentially increasing susceptibility to ATDs. Based on thyroid functional status and antibody profiles, MG-associated ATDs can be classified into three principal clinical subtypes: Graves' disease presenting with hyperthyroidism, Hashimoto's thyroiditis (HT) characterized by hypothyroidism and euthyroid individuals exhibiting solely thyroid antibody (TAb) positivity. Immunologically, the characteristic autoantibody spectrum in ATDs primarily comprises thyroid-stimulating hormone receptor antibodies (TRAb), thyroid peroxidase antibodies (TPOAb), and thyroglobulin antibodies (TgAb). Clinical studies demonstrate that MG patients with positive thyroid antibodies exhibit significantly higher serum levels of acetylcholine receptor antibodies (AChRAb) compared to antibody-negative individuals, accompanied by marked T-cell abnormalities [10]. Furthermore, serum AChRAb levels show a linear correlation with TRAbs in MG patients, suggesting potential immunological cross-reactivity between AChR and Thyroid-Stimulating Hormone(TSH)receptor epitopes [11]. Notably, as key biomarkers of ATDs, TgAb and TPOAb levels are closely associated with HT. Importantly, the prevalence of TgAb and TPOAb positivity is significantly higher in MG patients than in relatively healthy populations [12], indicating their potential involvement in MG pathogenesis, although the precise mechanisms require further investigation.

Studies demonstrate that approximately 16 % of MG patients exhibit thyroid dysfunction, while the prevalence of abnormal thyroid antibodies reaches 70 % - significantly higher than those with functional abnormalities. Notably, 56 % of MG patients exhibit isolated TAb positivity with normal thyroid function, indicating potential subclinical autoimmune dysregulation that may be pathophysiologically linked to MG [7,13,14]. Clinically relevant is the observation that patients with low-titer thyroid antibodies may remain asymptomatic despite developing hypothyroidism. Of particular concern, these individuals demonstrate significantly elevated risk for generalized myasthenic crisis, with the antibody-positive euthyroid subgroup being especially vulnerable to clinical oversight due to their asymptomatic presentation, necessitating heightened clinical awareness. Current research predominantly focuses on MG patients with thyroid dysfunction, particularly those with hyperthyroidism (Graves' disease). While existing literature has primarily investigated TRAb in MG patients with thyroid autoimmunity, studies on TgAb and TPOAb remain limited. Current evidence confirms that TgAb and TPOAb, recognized as sensitive serological markers for HT, predict an elevated risk of future hypothyroidism, which may subsequently contribute to worsened MG severity and poorer clinical outcomes. This study conducted a comprehensive retrospective analysis of clinical data from MG patients with abnormal TPOAb/TgAb levels, systematically comparing their clinical characteristics and investigating potential associations with disease progression. Our findings elucidate the pathophysiological roles of these thyroid antibodies in MG and provide clinically actionable evidence to guide therapeutic decision-making.

## 2 Methods

### 2.1 Study design

This retrospective, single-center, cross-sectional study analyzed MG patients treated at the First Affiliated Hospital of Dalian Medical University between January 1, 2022, and December 31, 2024. The study protocol was approved by the Institutional Ethics Committee of the First Affiliated Hospital of Dalian Medical University (Approval No.: PJ-KS-KY-2024-700).

### 2.2 Participants

#### 2.2.1 Inclusion criteria

(1) Age ≥18 years; (2) Definitive MG diagnosis according to established criteria [2], requiring: (i) characteristic clinical manifestations (fluctuating skeletal muscle weakness worsening with activity and improving with rest, plus positive fatigue/ice pack test); AND (ii) at least one of the following: (a) positive neostigmine test, (b) abnormal neurophysiological findings (low-frequency Repetitive Nerve Stimulation(RNS)showing decremental response or increased jitter on SFEMG), or (c) seropositivity for AChRAb or MuSKAb.

#### 2.2.2 Exclusion criteria

Patients were excluded if they met any of the following conditions: (1) age <18 years; (2) MG with concomitant organ-specific autoimmune diseases other than ATDs; (3) presence of systemic comorbidities including pregnancy, severe dysfunction of major organs (heart, lungs, liver, or kidneys), or malignancy; (4) MG with severe psychiatric disorders; (5) history of thyroid disease or thyroid surgery, or current use of antithyroid medications/thyroid hormone replacement therapy; (6) abnormal thyroid function tests (defined as any abnormality in TSH, FT3, or FT4).

### 2.3 Data collection

Patient data were extracted from the Hospital Information System (HIS) of the First Affiliated Hospital of Dalian Medical University, including: (1) baseline characteristics (age at onset, sex, primary/recurrent, initial symptoms, disease duration, MGFA classification and clinical scores); (2) laboratory results (TSH, FT3, FT4, TPOAb, TgAb, and MG-specific antibodies); (3) diagnostic findings (thymic CT/MRI, repetitive nerve stimulation); and (4) treatment regimens and medication details. For patients receiving glucocorticoids or other immunosuppressants at baseline, we documented the specific drug, daily dosage (converted to prednisone equivalents where applicable), and total treatment duration before study enrollment. However, due to the retrospective nature of this study, detailed records regarding dose tapering schedules were not systematically available across the cohort.

### 2.4 Statistical analysis

All data were processed and analyzed using SPSS 25.0. Categorical variables are presented as percentages (%) and compared using the chi-square test, with a P-value <0.05 considered statistically significant. Continuous variables were assessed for normality with the Kolmogorov-Smirnov test. Normally distributed data are presented as mean ± standard deviation (SD) and compared using paired t-tests (for intragroup comparisons), independent t-tests (for two groups), or one-way ANOVA (for multiple groups). Non-normally distributed data are presented as median and interquartile range (IQR) and compared using the Wilcoxon signed-rank test (intragroup), Mann-Whitney *U* test (two independent groups), or Kruskal-Wallis H test (multiple groups). For one-way ANOVA and Kruskal-Wallis H tests showing significance, post-hoc pairwise comparisons were performed using the LSD test or with Bonferroni correction, where an adjusted P < 0.05 indicated statistical significance. To assess the independent influence of the TPOAb + TgAb + status on treatment response, a multivariate logistic regression analysis was performed. The model adjusted for potential confounders, including age at onset, sex, relapse status, thymoma, and baseline MGFA classification. For the purpose of multivariate analysis, the baseline MGFA classification was dichotomized into mild (Class I, II) and moderate-severe (Class III, IV, V) groups.

## 3 Results

### 3.1 Patient characteristics

**Fig. 1:**
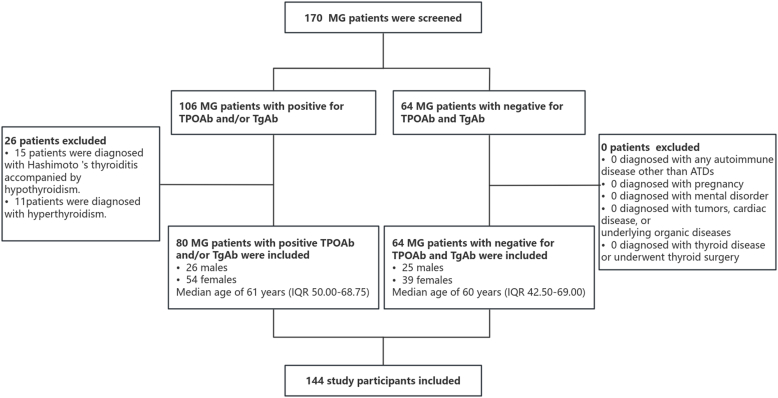
Study Flowchart showing the selection process for patients with MG. A total of 170 patients with MG were screened. Of these, 26 were excluded: 15 due to comorbid Hashimoto's thyroiditis with hypothyroidism and 11 due to hyperthyroidism. Finally, 144 patients were enrolled and stratified into a thyroid antibody-positive group (defined as positive for TPOAb and/or TgAb, n = 80) and a negative group (n = 64).

This retrospective study initially identified 170 MG patients treated at the First Affiliated Hospital of Dalian Medical University between January 1, 2022 and December 31, 2024. The cohort comprised 106 TAb+ (TPOAb and/or TgAb positive) and 64 TAb- (TPOAb and TgAb negative) MG patients. Following exclusion of 11 patients with concurrent hyperthyroidism receiving antithyroid treatment and 15 patients with hypothyroidism secondary to Hashimoto's thyroiditis, a final sample of 144 patients was included for comprehensive analysis (Fig. 1).

Among the 144 MG patients analyzed, 93 (64.6 %) were female, with a median onset age of 61 years (IQR 50.00–68.75) and median disease duration of 0.5 years (IQR 0.10–4.00). TAb + MG (n = 80, 55.6 %) constituted the majority of the cohort. Comparative analysis revealed no significant differences among TAb+, TAb-, and All-MG groups regarding onset age, sex distribution, disease duration, initial symptoms, disease course (long/short), episode type (primary/recurrent), onset timing (early/late), baseline medications, or QMG/MG-ADL scores pre- and post-treatment. However, TAb + MG demonstrated significantly higher baseline MGFA classification and AChRAb seropositivity rates (P = 0.013, Table 1).

**Table 1:** Clinical Features of TAb + MG, TAb-MG, and All-MG patients.

	All-MG (n = 144)	TAb + MG(n = 80)	TAb-MG(n = 64)	P
Gender (n, %)				
Male	51(35.4)	26(32.5)	25(39.1)	0.328
Female	93(64.6)	54(67.5)	39(60.9)	
Age (years, Median ± IQR)	61.00 (48.00, 69.00)	61.00 (50.00, 68.75)	60.00 (42.50, 69.00)	0.241
Duration (years, Median ± IQR)	0.50 (0.10, 4.00)	0.50 (0.10, 4.00)	0.60 (0.20, 4.00)	0.624
Symptoms (n, %)				
Extraocular muscles	88(61.1)	48(60.0)	40(62.5)	0.932
Extremities	21(14.6)	13(16.3)	8(12.5)	
Medulla oblongata muscle	31(21.5)	17(21.3)	14(21.9)	
Respiratory muscle	4(2.8)	2(2.5)	2(3.1)	
Short Disease Duration (n, %)	73(50.7)	41(51.2)	32(50.0)	0.881
Long Disease Duration (n, %)	71(49.3)	39(48.8)	32(50.0)	
Initial (n, %)	66(45.8)	34(42.5)	32(50.0)	0.369
Relapsed (n, %)	78(54.2)	46(57.5)	32(50.0)	
EOMG (n, %)	43(29.9)	22(27.5)	21(32.8)	0.489
LOMG (n, %)	101(70.1)	58(72.5)	43(67.2)	
Baseline MGFA class at screening (n, %)				
Ⅰ	56(38.9)	35(43.8)	21(32.8)	0.045^a^
Ⅱ	43(29.9)	16(20.0)	27(42.2)	
Ⅲ	28(19.4)	19(23.8)	9(14.1)	
Ⅳ	14(9.7)	9(11.3)	5(7.8)	
Ⅴ	3(2.1)	1(1.3)	2(3.1)	
Clinical scores at baseline(Median ± IQR)				
QMG	9.50 (6.00, 15.00)	9.50 (5.25,15.75)	9.50 (6.00, 13.75)	0.989
MG-ADL	6.00 (4.00,9.00)	6.00 (4.00,9.00)	6.00 (4.00,8.00)	0.443
Clinical scores after treatment(Median ± IQR)				
QMG	8.00 (5.00,12.00)	8.00 (3.25,12.00)	8.00 (5.00,11.00)	0.659
MG-ADL	5.00 (3.00, 6.75)	5.00 (3.00,7.00)	4.50 (3.00,6.00)	0.720
Myasthenia gravis therapy at baseline (n, %)				0.246
PB therapy and no medication	107(74.3)	63(78.8)	44(68.8)	
Any Steroid	15(10.4)	9(11.3)	6(9.4)	
Treatment duration				0.519
Recent use (≤4 weeks)	9(6.2)	6(7.5)	3(4.7)	
Long-term use (>4 weeks)	4(4.2)	3(3.8)	3(4.7)	
Median daily prednisone-equivalent dose, mg (IQR)	25.00(15.00,35.00)	30.00(18.75,45.00)	17.50(8.75,35.00)	0.153
Any NSISTs	7(4.9)	2(2.5)	5(7.8)	
Combination therapy	15(10.4)	6(7.5)	9(14.1)	
With thymoma (n, %)	37(25.7)	23(28.7)	14(21.9)	0.348
RNS positive (n, %)	85(59.0)	52(65.0)	33(51.6)	0.103
Antibodies (n, %)				
AchRAb positive	124(86.1)	74(92.5)	50(78.1)	0.013^a^
MuSKAb positive	4(2.8)	2(2.5)	2(3.1)	0.821
TitinAb positive	34(23.6)	20(25.0)	14(21.9)	0.661
RyRAb positive	18(12.5)	12(15.0)	6(9.4)	0.310

Note: Data are median(interquartile range)or n(%). MG-ADL = Myasthenia Gravis Activities of Daily Living. MGFA = Myasthenia Gravis Foundation of America. QMG = Quantitative Myasthenia Gravis score.

PB=Pyridostigmine bromide. Steroid = glucocorticoid (including prednisone, methylprednisolone, etc.). NSIST = non-steroidal immunosuppressant therapy.

a Definitions: All-MG = all myasthenia gravis (MG) patients in this study; TAb + MG = positive thyroid peroxidase antibody (TPOAb) and/or thyroglobulin antibody (TgAb); TAb-MG = MG patients for both TPOAb and TgAb are negative. Early-onset MG (EOMG): disease onset at age ≤50 years; late-onset MG (LOMG): onset >50 years. Short disease duration (SDD): ≤0.5 years; long disease duration (LDD): >0.5 years.

### 3.2 Clinical features of TPOAb/TgAb subgroups

**Fig. 2:**
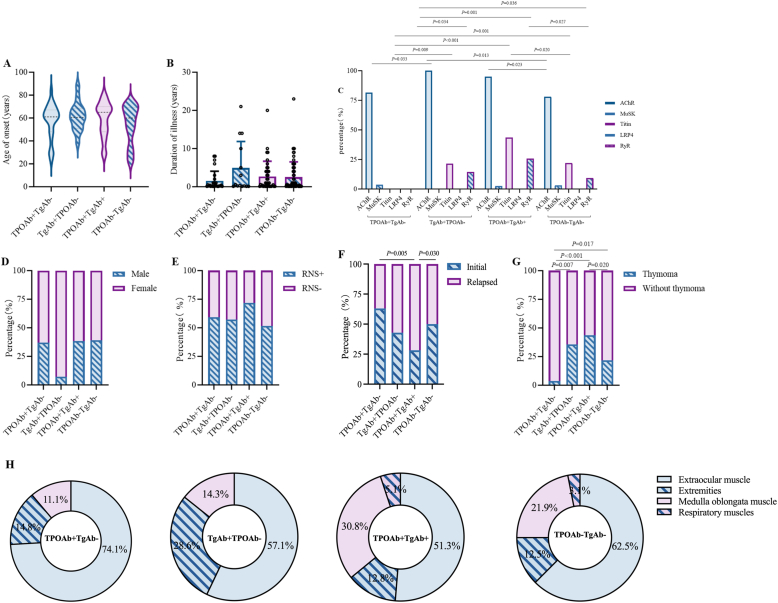
Clinical Features of MG by Thyroid Antibody Subgroups. **(A)** Age at onset distribution across MG subgroups (Mann-Whitney test). **(B)** Disease duration patterns (Mann-Whitney test). **(C)** Antibody profiles revealed significantly higher Titin/RyR antibody positivity in TPOAb + TgAb + MG and predominant AChR antibody seropositivity in TgAb + TPOAb-patients (χ^2^ test). **(D)** Sex distribution (χ^2^ test). **(E)** Repetitive nerve stimulation results (χ^2^ test). **(F)** Disease recurrence patterns showing higher relapse rates in TPOAb + TgAb + MG (χ^2^ test). **(G)** Thymoma association demonstrated low prevalence in TPOAb + TgAb- MG versus high frequency in TPOAb + TgAb + MG (χ^2^ test). **(H)** Initial symptom patterns with ocular myasthenia predominance in TPOAb + TgAb- MG and bulbar involvement in TPOAb + TgAb + MG(χ^2^ test).

Based on TAb profiles, TAb + MG patients were stratified into three subgroups: TPOAb + TgAb- (n = 27), TgAb + TPOAb- (n = 14), and TPOAb + TgAb+ (n = 39), which were compared with TAb- MG (n = 64) in a four-group analysis. The analysis revealed no significant intergroup differences in onset age (Fig. 2A), disease duration (Fig. 2B), sex distribution (Fig. 2D), or RNS positivity rates (Fig. 2E). Significant intergroup differences were observed in three key aspects: However, distinct immunological and clinical patterns emerged: (1) TPOAb + TgAb- MG showed the lowest TitinAb and RyRAb positivity; (2) TgAb + TPOAb- MG demonstrated the highest AChRAb positivity; and (3) TPOAb + TgAb + MG exhibited the highest TitinAb and RyRAb positivity (all P < 0.05, Fig. 2C). Clinically, TPOAb + TgAb + MG had significantly higher relapse rates (Fig. 2F) and thymoma prevalence, whereas TPOAb + TgAb- MG rarely presented with thymoma (Fig. 2G). Although not statistically significant, initial symptom patterns differed, with TPOAb + TgAb- MG predominantly showing ocular myasthenia and TPOAb + TgAb + MG more frequently exhibiting bulbar involvement (Fig. 2H).

### 3.3 Associations of clinical features with TPOAb/TgAb titers

**Fig. 3:**
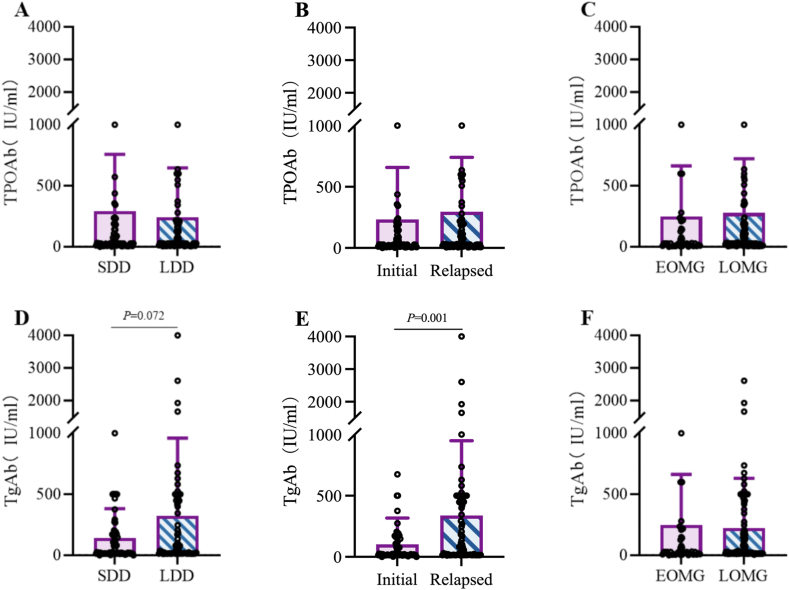
Clinical Features and Thyroid Antibody Titers. **The Y-axis is truncated, omitting values within the 1000**–**1500 range. (A、D)** TPOAb and TgAb titer comparisons stratified by disease duration (long/short; Mann-Whitney test). 19 data points (13 %) are excluded in Panel A, and 2 (1.4 %) in Panel D. **(B、E)** Antibody titer analysis between initial and relapsed MG, demonstrating significantly elevated TgAb titers in recurrent MG (Mann-Whitney test). Exclusions: 19 (13 %) in Panel B, 2 (1.4 %) in Panel E.**(C、F)** Titer comparisons by age of onset (early/late-onset; Mann-Whitney test), with 19 (13 %) and 6 (4.2 %) data points excluded in Panels C and F, respectively.

When stratifying patients by disease duration (long/short), episode type (initial/relapsed), and onset age (early/late), we observed: (1) TPOAb titers showed no significant differences across all stratification criteria (Fig. 3A–C); (2) TgAb titers were numerically higher (though not statistically significant) in the long-duration group versus short-duration (Fig. 3D), significantly elevated in recurrent cases compared to initial episodes (P < 0.05, Fig. 3E), and comparable between early- and late-onset MG (Fig. 3F).

### 3.4 Treatment response across TPOAb/TgAb subgroups

**Fig. 4:**
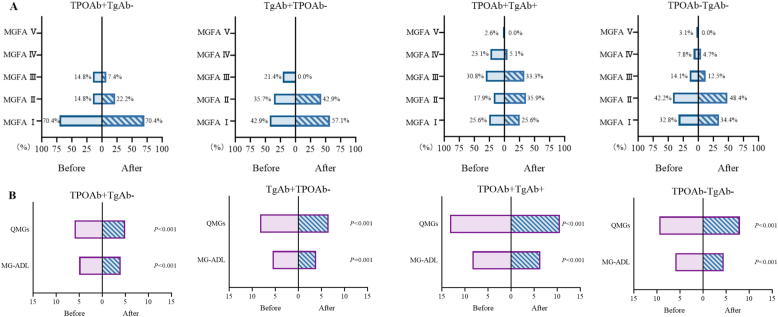
Treatment Response across Thyroid Antibody Subgroup. **(A)** Post-treatment MGFA improvement was significant in TgAb + TPOAb- (P = 0.025), TPOAb + TgAb+ (P < 0.001), and TPOAb-TgAb- (P = 0.001) groups (Wilcoxon signed-rank test). **(B)** All four subgroups showed significant reductions in both QMG and MG-ADL scores, with TPOAb + TgAb- and TPOAb-TgAb- analyzed by Wilcoxon signed-rank test, while TgAb + TPOAb- and TPOAb + TgAb + groups were evaluated using paired t-tests.

Comparative analysis of baseline MGFA classification revealed distinct patterns: TPOAb + TgAb- MG predominantly presented with MGFA I, whereas TPOAb + TgAb + MG were more frequently classified as MGFA III. Post-treatment MGFA improvement was observed in all subgroups except TPOAb + TgAb- MG (P = 0.157, Fig. 4A). Although all groups showed significant reductions in both QMG and MG-ADL scores following treatment, TPOAb + TgAb + MG maintained higher residual scores (QMG: 10.59; MG-ADL: 6.36) compared to other subgroups (P < 0.001), indicating greater disease severity and suboptimal response to conventional therapy (Fig. 4B).

### 3.5 Differential therapeutic efficacy among TPOAb/TgAb subgroups

**Fig. 5:**
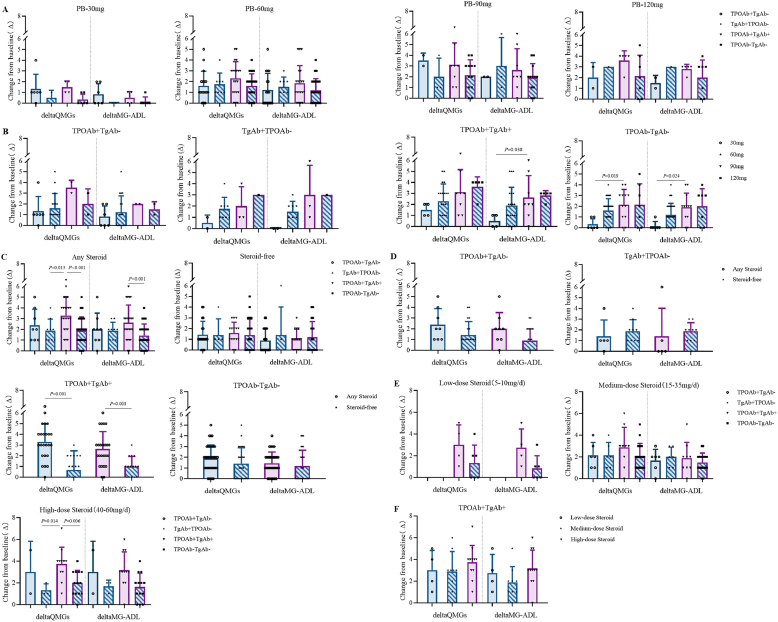
Therapeutic Efficacy among Thyroid Antibody Subgroups. Any Steroid: indicates patients receiving any glucocorticoid regimen; Steroid-free: denotes patients without glucocorticoid treatment. **Treatment Response Analysis with Truncated Y-axis (6–7 range excluded). (A)** Pyridostigmine bromide (PB) dose-response analysis revealed potential sampling bias in TgAb + TPOAb- (PB30mg: n = 2; PB120mg: n = 1) and TPOAb + TgAb- (PB90mg: n = 2; PB120mg: n = 2) subgroups due to limited sample size, analyzed by Kruskal-Wallis test (PB30/60 mg) and one-way ANOVA (PB90/120 mg). **(B)** While TPOAb + TgAb- and TgAb + TPOAb- MG were ineligible for statistical analysis (insufficient samples), TPOAb + TgAb+ and TPOAb-TgAb- MG showed optimal response to PB90mg (Kruskal-Wallis with Bonferroni-corrected ANOVA). **(C)** Corticosteroid treatment demonstrated significant efficacy in TPOAb + TgAb + MG (one-way ANOVA for treated vs. Kruskal-Wallis for untreated MG). **(D)** TPOAb + TgAb + MG exhibited superior response to corticosteroid therapy versus untreated controls (Mann-Whitney test; 3 datapoints [7.7 %] excluded). **(E)** High-dose corticosteroids showed enhanced efficacy in TPOAb + TgAb + versus TPOAb-TgAb- MG (Bonferroni-corrected ANOVA/LSD test), though low-dose analysis was precluded in TPOAb + TgAb-/TgAb + TPOAb- MG (n = 0). **(F)** Dose-dependent effects within TPOAb + TgAb + MG were analyzed by one-way ANOVA.

All 142 MG patients (98.6 %) received pyridostigmine treatment, stratified into four dosage groups (30, 60, 90, and 120 mg/day). While no significant differences in clinical improvement were observed across TAb subgroups at these dosages (Fig. 5A), dose-response patterns emerged in specific subgroups: the 90 mg/day regimen demonstrated superior efficacy compared to 30 mg/day (P = 0.038) and 60 mg/day (P = 0.024) in both TPOAb + TgAb + MG and TPOAb-TgAb- MG, with comparable effectiveness to 120 mg/day (Fig. 5B). Patients were stratified by corticosteroid use into treatment and non-treatment groups. TPOAb + TgAb + MG demonstrated significant clinical improvement with corticosteroids (P = 0.001, Fig. 5C). Comparative analysis revealed superior treatment response in steroid-treated versus untreated TPOAb + TgAb + MG (P = 0.001), while no significant differences were observed in the other three subgroups (Fig. 5D). Further stratification by dosage (low-dose: 5–10 mg/day, medium-dose: 15–35 mg/day, high-dose: 40–60 mg/day) showed that high-dose corticosteroids elicited better responses in TPOAb + TgAb + MG compared to TPOAb-TgAb- MG (P = 0.006, Fig. 5E), though no significant dose-dependent effects were detected within TPOAb + TgAb + MG itself (Fig. 5F).

### 3.6 Multivariate analysis of predictors for high-dose glucocorticoids response

To determine whether the superior response to high-dose glucocorticoids observed in the TPOAb + TgAb + MG was independent of its associated clinical features, a multivariate logistic regression model was constructed. The analysis included the TPOAb + TgAb + status (as a binary variable compared to all other patients), thymoma, relapse status, age at onset, sex, and baseline MGFA classification. The model revealed that the TPOAb + TgAb + status itself was not an independent predictor of treatment response (OR = 0.077, 95 % CI: 0.000–23.487; P = 0.380). None of the other adjusted covariates demonstrated statistical significance. The model indicated a perfect separation of data points for the baseline MGFA variable, rendering its coefficient estimate unreliable, it was therefore not interpreted. This model suggests that the association between TPOAb + TgAb + status and response to high-dose glucocorticoids may be influenced by its associated clinical features (Table 2).

**Table 2:** Multivariate logistic regression analysis of factors associated with treatment response to high-dose glucocorticoids.

Variable	OR (95 % CI)	P
TPOAb + TgAb + status	0.077 (0.000–23.487)	0.380
Thymoma	1.082 (0.020–59.995)	0.969
Relapse status	16.531 (0.651–419.542)	0.089
Age at onset	1.043 (0.964–1.128)	0.295
Female sex	0.039 (0.001–1.490)	0.081
Baseline MGFA	N/A	0.998

Note:OR = odds ratio; CI = confidence interval.

The model assessed predictors for favorable response to high-dose glucocorticoids (40–60 mg/day). TPOAb + TgAb + status was coded as a binary variable (positive vs. all other patients).

The coefficient for the baseline MGFA variable (dichotomized) is not shown due to complete or quasi-complete separation of data points, which resulted in an unreliable estimate.

This study elucidates the significant clinical implications of TPOAb/TgAb positivity in patients with myasthenia gravis (MG). We identified a distinct TPOAb + TgAb + MG subgroup characterized by a "triple-high" profile (high disease severity, high relapse rate, and high thymoma comorbidity), which demonstrates superior responsiveness to high-dose glucocorticoid therapy (40–60 mg/day). Furthermore, TgAb levels may serve as a potential biomarker for monitoring disease activity. These findings provide critical evidence to guide precision medicine in MG management.

## 4 Discussion

The comorbidity of TPOAb/TgAb and MG suggests shared immunopathological pathways. Our stratified analysis reveals that the distinct clinical features of TPOAb + TgAb + MG (including disease severity and treatment resistance) may stem from cross-reactive immune responses between TAb and NMJ antigens, or Fc receptor-mediated antibody-dependent cellular cytotoxicity (ADCC) exacerbating disease progression [14]. While previous studies reported high TAb prevalence in MG [13], their impact on clinical and therapeutic significance remains unclear. Importantly, we demonstrate that double-positive TPOAb + TgAb + status correlates with higher Titin/RyR antibody seropositivity and increased relapse risk, suggesting a potential interactions between thyroid autoimmunity and MG antibody profiles/disease progression. TPOAb/TgAb testing not only facilitates MG subgroup classification but also may provide novel disease activity markers (for instance, rising TgAb titers during relapsed). For TPOAb + TgAb + MG, early initiation of targeted biologics could potentially improve outcomes.

Our data demonstrate that approximately 55.6 % of MG patients exhibit TAb positivity while maintaining normal thyroid function, a finding consistent with previous clinical observations reported by Ji Wenzhen's team in China [13]. Further analysis revealed that TAb + MG patients showed higher AChRAb seroprevalence compared to TAb- MG, suggesting potential epitope sharing between thyroid antigens and AChR that may lead to cross-reactive immune responses. These results indicate a potential immunopathological linkage between thyroid autoantibodies and pathogenic antibodies targeting the postsynaptic membrane of the NMJ. To further elucidate the clinical significance of TAb in MG, we systematically analyzed the clinical characteristics of MG patients stratified by thyroid antibody profiles (TPOAb/TgAb).

Our findings demonstrate that the TPOAb + TgAb- MG phenotype predominantly manifests as ocular myasthenia gravis (OMG). Notably, thyroid peroxidase (TPO) is expressed in orbital tissues of patients with thyroid-associated orbitopathy (TAO) [15], with TPOAb levels correlating with orbital involvement severity [16,17]. In TAO, activated autoreactive T cells stimulate glycosaminoglycan (GAG) production by fibroblasts through cytokine release (IFN-γ, TNF-α), leading to extraocular muscle hypertrophy and tissue edema [18]. OMG, representing the most common MG phenotype (85 % of cases), presents with characteristic ptosis and diplopia [19], features attributable to the unique immunological and physiological properties of extraocular muscles: specialized postsynaptic membrane structure [20], persistent expression of highly immunogenic γ-subunits with elevated AChR density, and deficient complement regulation [18]. Importantly, extraocular muscles show preferential involvement in both AChRAb-positive and TPOAb-positive MG patients, suggesting potential epitope sharing between AChR and TPO. While strongly associated with OMG, TPOAb + TgAb- MG exhibits significantly reduced thymoma incidence. This clinical distinction may reflect a Th1-biased immune response, where IFN-γ promotes TPOAb production while suppressing Th17 differentiation, potentially preventing the Th17/Treg imbalance characteristic of thymoma [21,22]. Strikingly, thymoma-associated MG (TAMG) demonstrates high seropositivity for striational antibodies (TitinAb, RyRAb) [23], whereas TPOAb + TgAb- MG patients show markedly lower positivity, suggesting thymoma absence may limit aberrant muscle antigen exposure and subsequent autoimmunity. It is tempting to speculate that the reduced thymoma incidence and lower TitinAb/RyRAb positivity in TPOAb + TgAb- MG might be linked to an IFN-γ-dominant immune milieu, which could potentially suppress Th17 differentiation. However, this potential mechanism requires direct validation in future studies.

In the present study, TgAb + TPOAb- MG patients demonstrated significantly elevated AChRAb positivity. This clinical association is supported by genetic evidence linking both TgAb and AChRAb to the ∗HLA-DRB1∗03:01 (HLA-DR3) allele [24,25]. Given the established role of HLA class II molecules in mediating T-cell selection in the thymus and antigen presentation to CD4^+^ T cells in the periphery [26], this shared HLA association suggests that the HLA-DR3 allele may drive a unified immunopathogenic mechanism by perturbing these fundamental functions. We speculate that this mechanism likely stems from the synergy between aberrant thymic selection of autoreactive T cells and peripheral cross-antigen presentation, culminating in the concurrent loss of immune tolerance to both Tg and AChR. This hypothesis, while consistent with the genetic and clinical data, remains speculative and highlights the need for experimental confirmation of the precise cellular and molecular events. Furthermore, the observed positive correlation between TgAb titers and MG disease activity suggests TgAb might serve as a potential biomarker for assessing disease activity. Both the proposed mechanism and the biomarker potential of TgAb warrant rigorous validation in future multicenter, large-scale prospective studies.

TPOAb + TgAb + MG exhibit a characteristic 'triple-high' profile: severe disease progression, high thymoma incidence, and elevated specific antibody positivity, with poor response to conventional therapy. Clinically, this subgroup predominantly presents as generalized MG (MGFA III) with relatively severe symptoms. A key clinical observation from our study is that this subgroup demonstrated a superior response to high-dose corticosteroids (40–60 mg/day) compared to the TPOAb-TgAb- MG. However, multivariate logistic regression analysis revealed that the TPOAb + TgAb + status itself was not an independent predictor of this favorable treatment outcome. This crucial finding necessitates a reinterpretation: the apparent benefit from high-dose glucocorticoids is likely not a direct effect of the double-positive antibody status per se, but reflects the attributes of the distinct clinical subgroup identified by this serological marker. This subgroup aggregates features of heightened disease activity, such as significantly increased relapse rates and thymoma prevalence. Consequently, the clinical utility of the TPOAb + TgAb + profile lies in its ability to identify—serving as a powerful clinical tool to pinpoint patients who, due to their high disease activity, both necessitate and are positioned to benefit from intensive immunosuppressive therapy.

The pathophysiological basis of this subgroup may involve several interconnected mechanisms. As hallmark antibodies of HT, TPOAb and TgAb can directly mediate thyroid tissue damage through complement activation and ADCC [14]. The resultant subclinical hypothyroidism or the broader systemic autoimmune diathesis itself may exacerbate MG symptoms by causing dysregulation of muscle protein metabolism, impairing mitochondrial energy production, and altering neuromuscular transmission [27,28]. Furthermore, the systemic autoimmune state marked by these antibodies is often accompanied by a proinflammatory milieu, characterized by elevated cytokines such as IL-6 and IL-17 [29,30]. These cytokines can intensify inflammation at the neuromuscular junction (NMJ) and promote abnormal thymic epithelial cell proliferation [31,32], thereby creating a feed-forward loop that amplifies both diseases. Beyond these effects, the significant comorbidity suggests shared immunogenetic susceptibility, particularly involving specific MHC class II polymorphisms, which may also underpin the high thymoma incidence observed in this subgroup. Within the abnormal thymic microenvironment, autoreactive B cells may receive sustained activation signals against both neuromuscular and thyroid antigens, thereby perpetuating a vicious cycle of autoantibody production [33]. Finally, the high seropositivity for TitinAb and RyRAb—antibodies highly specific for TAMG—further solidifies the link between this distinct thyroid autoantibody profile, underlying thymic pathology, and the overall severity of the disease.

While this study provides novel insights into the clinical characteristics and treatment of TPOAb/TgAb-positive MG, several limitations should be acknowledged. First, as a single-center retrospective study with a limited sample size, particularly in the high-dose steroid subgroup, our statistical power for multivariate modeling was constrained. Second, although we observed a strong association between TgAb titers and relapse, the lack of longitudinal titer data precludes an analysis of dynamic changes. Future studies incorporating serial measurements are warranted to assess its utility as a disease activity biomarker. Third, the study design did not incorporate thyroid ultrasound examinations. Future research correlating imaging features with autoantibody levels could provide a more comprehensive pathophysiological picture. Finally, although we systematically compared responses to pyridostigmine and glucocorticoids, the study did not assess other interventions including intravenous immunoglobulin or non-steroidal immunosuppressants. Notwithstanding these limitations, our findings underscore the potential clinical value of the TPOAb + TgAb + serological profile in identifying a distinct MG subgroup, thereby laying the groundwork for future research into targeted immunosuppressive strategies for these patients.

## CRediT authorship contribution statement

**Hanyu Xin:** Conceptualization, Data curation, Formal analysis, Visualization, Writing – original draft, Writing – review & editing. **Mingou Lu:** Conceptualization, Data curation, Supervision, Validation, Writing – review & editing.

## Ethics statement

This study involving human participants was reviewed and approved by the Ethics Committee of the First Affiliated Hospital of Dalian Medical University (Approval No. PJ-KS-KY-2024-700). The research adhered to the principles outlined in the Declaration of Helsinki, ensuring the confidentiality and ethical treatment of all patient data.

## Consent for publication

All the authors agree to the publication of this study.

## Funding statement

This research did not receive any specific grant from funding agencies in the public, commercial, or not-for-profit sectors.

**Table:** List of Abbreviations

Abbreviation	Full Term
AChR	Acetylcholine receptors
ATDs	Autoimmune thyroid diseases
ADCC	Antibody-dependent cellular cytotoxicity
AChE	Acetylcholinesterase
BCR	B Cell Receptor
GAG	Glycosaminoglycan
HT	Hashimoto's thyroiditis
HIS	Hospital Information System
HLA-DR3	HLA-DRB1∗03:01
LRP4	Low-density lipoprotein receptor-related protein 4
MG	Myasthenia gravis
MuSK	Muscle-specific kinase
NMJ	Neuromuscular junction
OMG	Ocular myasthenia gravis
RyR	ryanodine receptors
RNS	Repetitive Nerve Stimulation
SD	Standard deviation
TAb	Thyroid antibody
TRAb	Thyroid-stimulating hormone receptor antibodies
TPOAb	Thyroid peroxidase antibodies
TgAb	Thyroglobulin antibodies
TSH	Thyroid-Stimulating Hormone
TAO	Thyroid-associated orbitopathy
TAMG	Thymoma-associated myasthenia gravis

## Declaration of competing interest

The authors declare that they have no known competing financial interests or personal relationships that could have appeared to influence the work reported in this paper.

## Data Availability

Data will be made available on request.
